# Behind the Counter

**Published:** 1893-03

**Authors:** 


### BEHIND THE COUNTER.

No. 3.

I have now held my place a whole week, and been fined only once, and that not severely. The girls say that is getting on very well for a raw hand. A queer customer from "ould Erin" elbowed her way to my counter. "Plaze mum, show me the hosses;" she said, addressing me. "The horses?" I repeated, enquiringly. "Shure, mum, the hosses—the hosses," she repeated with emphasis. It was some time before I could be made to understand that it was a clothes horse she desired to be shown, and I could'nt, for the life of me resist giggling in her face as I pointed the way to the wooden ware section, instead of a stable. Even the risibles of 13 were visibly excited, but that watchful monitor of female decorum, the floor-walker, singled me out for a first lesson at twenty-five cents tuition fee.

Thirteen has been very kind to me, and I have learned from her somewhat of her life-history, which I will recount in her own words. "I was the eldest of three sisters, favored with a pleasant home, indulgent parents and agreeable associates. My school days were passed with girls of nearly my own age, whose companionship was all that could be desired. At one of our school festivals I

formed the acquaintance of a young physician, whose attentions then and thereafter, became the subject of no little innocent raillery on the part of my associates. This coming to the ears of my parents, occasioned them no little concern, for aside from the question of diverting my mind from my studies, they had already formed plans for my future which an ill considered alliance would seriously interrupt. In short, I was peremptorily forbidden to receive the attentions of Dr. Fielding as a special visitor, but the doctor was a man not easily dissuaded from pursuing an object wherein his affections were centered. His insistence, which by no means displeased me, gained my consent to an occasional secret meeting which took on a hue of romance almost always welcomed in affairs of this nature, the discovery of which created a family commotion, ending in a breach which to this day has never been healed. I was not even allowed to choose between obedience to parental behests and open revolt, the former being sought to be enforced by physical restraint. At this I became desperate and was easily persuaded to an elopement, which terminated in a marriage with the doctor. From that eventful day the door of my girlhood home has been closed against me, but my married life was a calm and unusually happy one so long as good fortune permitted it to continue.

"The doctor never wavered in his kindly attentions. He rose to much eminence in his profession, and our home, though simple and void of ostentation was an exceedingly peaceful and contented one. The birth of a baby boy during the first year filled to the brim the measure of our happiness. He had attained to his ninth year, a strong healthy lad, well endowed, when a great sorrow clouded our lives. It has never left us. The doctor, in his capacity of Hospital surgeon in performing an autopsy received a slight wound in the wrist, through the awkwardness of an assistant. Little attention was paid to it at the time, but in less than one week he passed to the other life. It was a case of blood poisoning. We laid the form we had learned to love so well, now cold and tenantless, tenderly away with our buried hopes, never to revive in this world. The estate was not large, and it is my sole aim that it shall be no less when my darling boy shall be called upon to take upon himself the part of a man with all his manly life before him. It is for this that I am here, where I am accounted an old maid of most unsocial ways."

It was an idle day; the rain came down in drenching sheets, and the wild winds twisted and hurled it against the great show windows with fearful energy, and as my friend concluded her painful narrative, I clapsed her hand with a pressure that spoke to the heart as no words can speak, and great round tears gushed from her eyes and answered mine in the tenderest communings of the soul.

And so her life goes on from day to day, buoyed by a single hope that centres all her aims.

> "While memory watches o'er the sad review
> Of joys that faded like the morning dew." —PAULINE.

